# Supplementary material for: In Vivo Imaging and Kinetic Modeling of Novel Glycogen Synthase Kinase-3 Radiotracers [11C]OCM-44 and [18F]OCM-50 in Non-Human Primates
Source: Pharmaceuticals (Basel). 2023 Jan 28;16(2):194. doi: 10.3390/ph16020194 (PMC9959234; doi:10.3390/ph16020194)
Supplement: Supplementary file 1 [file pharmaceuticals-16-00194-s001.zip › pharmaceuticals-2116277-supplementary.pdf]

***In vivo* imaging and kinetic modeling of novel glycogen synthase kinase-3 radiotracers [^{11}C]OCM-44 and [^{18}F]OCM-50 in non-human primates**

Kelly Smart, Ming-Qiang Zheng, Daniel Holden, Zachery Felchner, Li Zhang, Yanjiang Han, Jim Ropchan, Richard E. Carson, Neil Vasdev, and Yiyun Huang
*Correspondence to richard.carson@yale.edu; neil.vasdev@utoronto.ca and henry.huang@yale.edu

SUPPLEMENTAL MATERIAL

Table S1. K_1 values for [^{11}C]OCM-44 (two-tissue compartment model, 60 min) and [^{18}F]OCM-50 (one-tissue compartment model, 120 min) in non-human primates.

**K_1 (mL·cm^{-3}·min^{-1})**	**[^{11}C]OCM-44**		**[^{18}F]OCM-50**
	animal 1	*animal 2*	
Frontal cortex	0.380	0.187	0.133
Temporal cortex	0.341	0.198	0.133
Occipital cortex	0.327	0.235	0.207
Cingulate	0.413	0.222	0.167
Insula	0.402	0.217	0.122
Caudate	0.412	0.269	0.102
Putamen	0.471	0.307	0.135
Pallidum	0.283	0.178	0.059
Hippocampus	0.297	0.229	0.096
Amygdala	0.280	0.229	0.083
Thalamus	0.308	0.239	0.102
Pons	0.251	0.248	0.126
Cerebellum	0.469	0.337	0.197

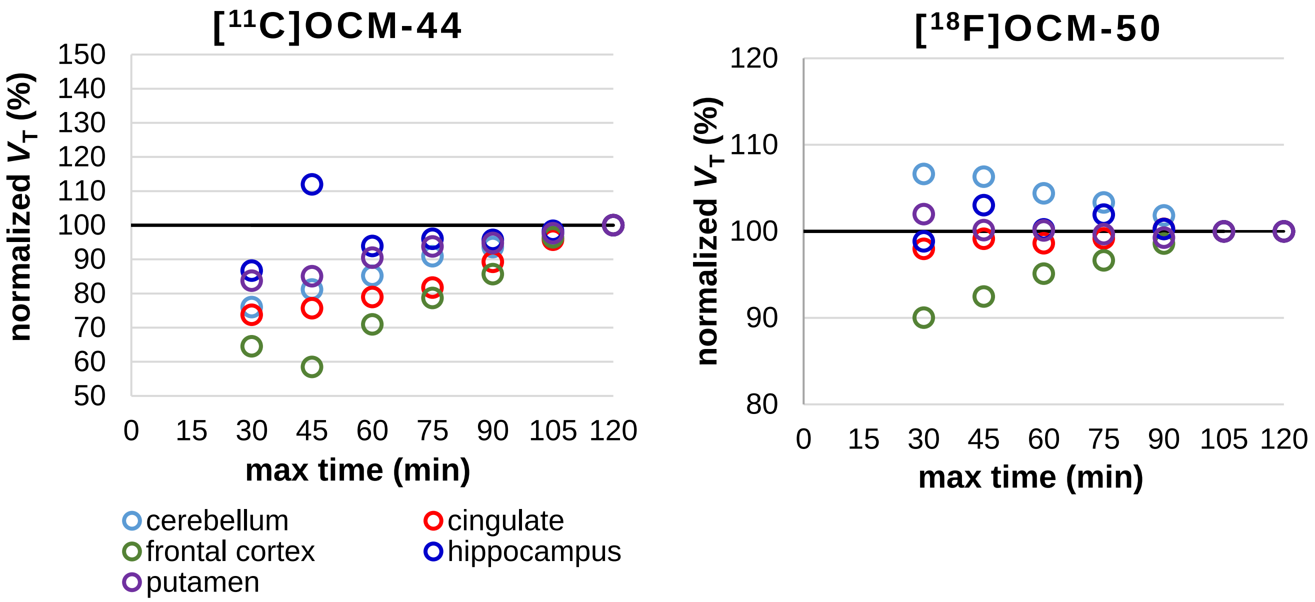

Fig. S1. Time stability of [^{11}C]OCM-44 and [^{18}F]OCM-50 V_T estimates. Left, [^{11}C]OCM-44 V_T values from 2TCM expressed as a percent of V_T from full 120-min scan data (mean of n=2). Right, [^{18}F]OCM-50 V_T values from 1TCM expressed as a percentage of V_T from full 120-min scan data (n=1, arterial samples were not available in the other baseline scan).

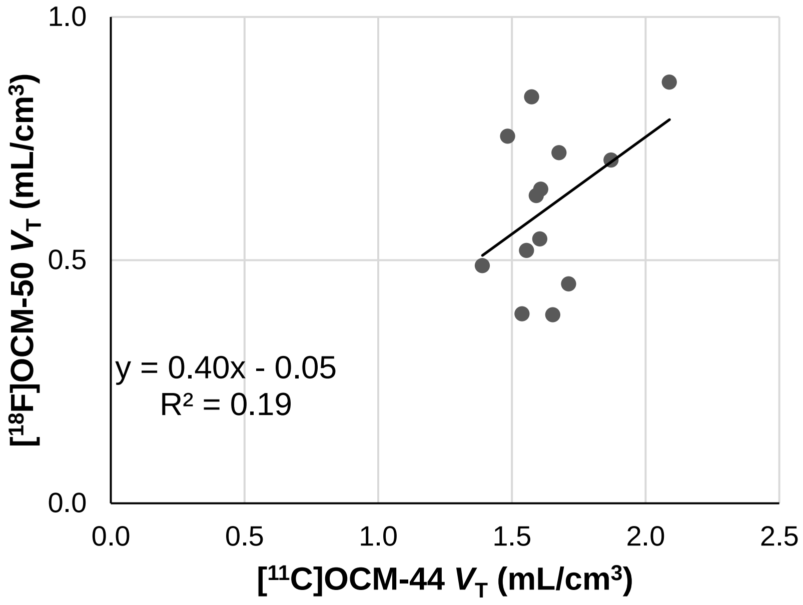

Fig. S2. Guo plot comparing V_T of [^{18}F]OCM-50 (1TCM, 120-min) to that of [^{11}C]OCM-44 (2TCM, 60-min) across brain regions. The predicted close linear relationship between binding availability of the two radiotracers was not apparent.
